# Supplementary material for: Biosynthesized Highly Stable Au/C Nanodots: Ideal Probes for the Selective and Sensitive Detection of Hg2+ Ions
Source: Nanomaterials (Basel). 2019 Feb 12;9(2):245. doi: 10.3390/nano9020245 (PMC6409943; doi:10.3390/nano9020245)
Supplement: Supplementary file 1 [file nanomaterials-09-00245-s001.pdf]

# Supplementary Materials

# Biosynthesized Highly Stable Au/C Nanodots: Ideal Probes for Selective and Sensitive Detection of $Hg^{2+}$ Ions

**Sada Venkateswarlu [1,†], Saravanan Govindaraju[2,4 †], Roopkumar Sangubotla[3], Jongsung Kim[3], Min-Ho Lee[4, *], Kyusik Yun[2, *]**

[1] Department of Nanochemistry, Gachon University, Gyeonggi-do 13120, Republic of Korea; venkisada67@gmail.com (S.V.)

[2] Department of Bionanotechnology, Gachon University, Gyeonggi-do, 13120, Republic of Korea; biovijaysaran@gmail.com (S.G.); ykyusik@gachon.ac.kr (K.Y)

[3] Department of Chemical and Biological Engineering, Gachon University, 1342 Seongnam Daero, Seongnam-Si, Gyeonggi-do, 13120, Republic of Korea. gachonroop@gmail.com (R.S); jongkim@gachon.ac.kr (J.K)

4 School of Integrative Engineering, Chung-Ang University, Seoul 06974, Republic of Korea; mhlee7@cau.ac.kr (M.H.L.)

† The authors are equally contributed

* Correspondence: mhlee7@cau.ac.kr (M.H.L.), ykyusik@gachon.ac.kr (K.Y)

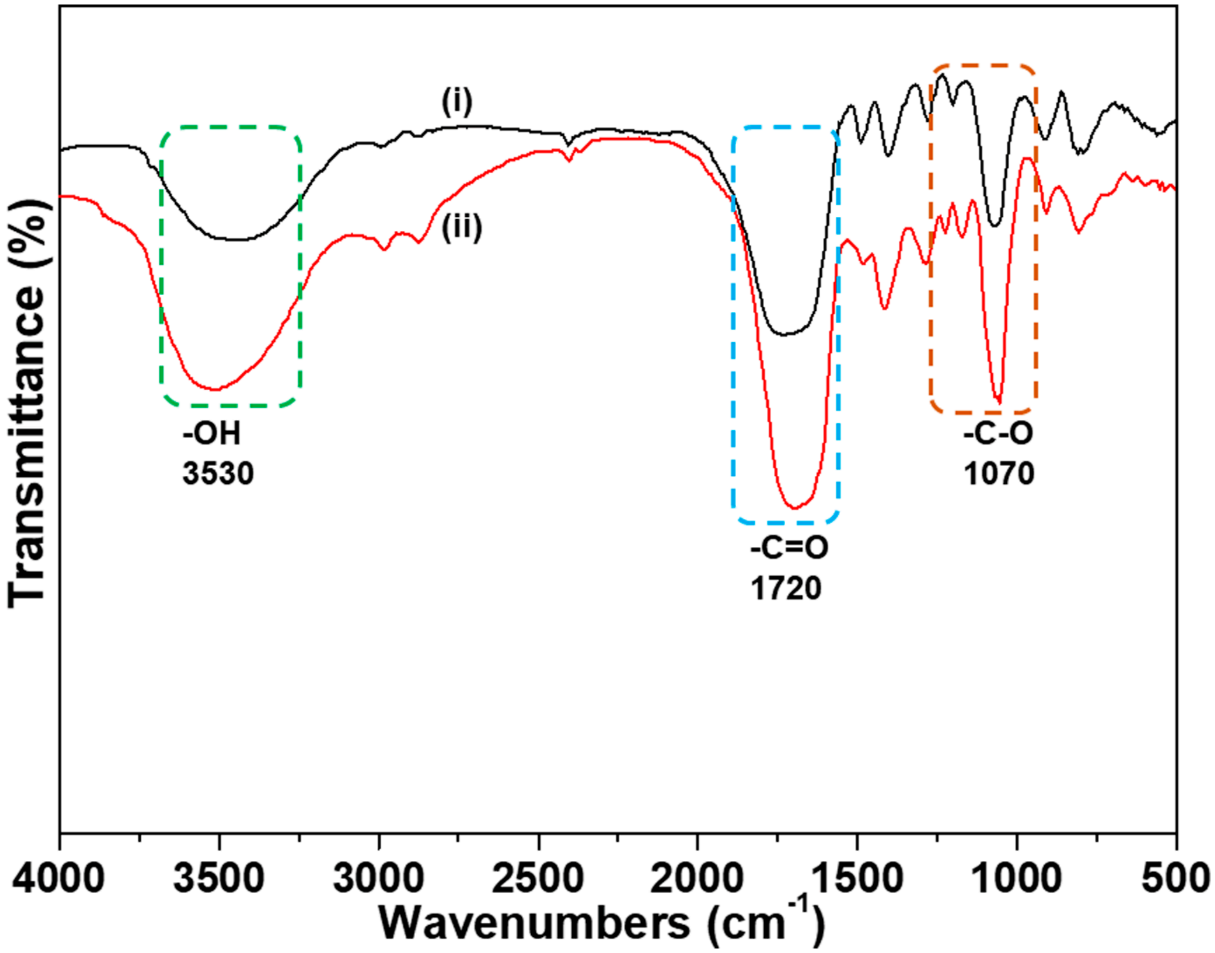


**Figure S1** FT-IR spectra of red onion leaves extract obtained from (i) Daegu and (ii) Incheon, South Korea.

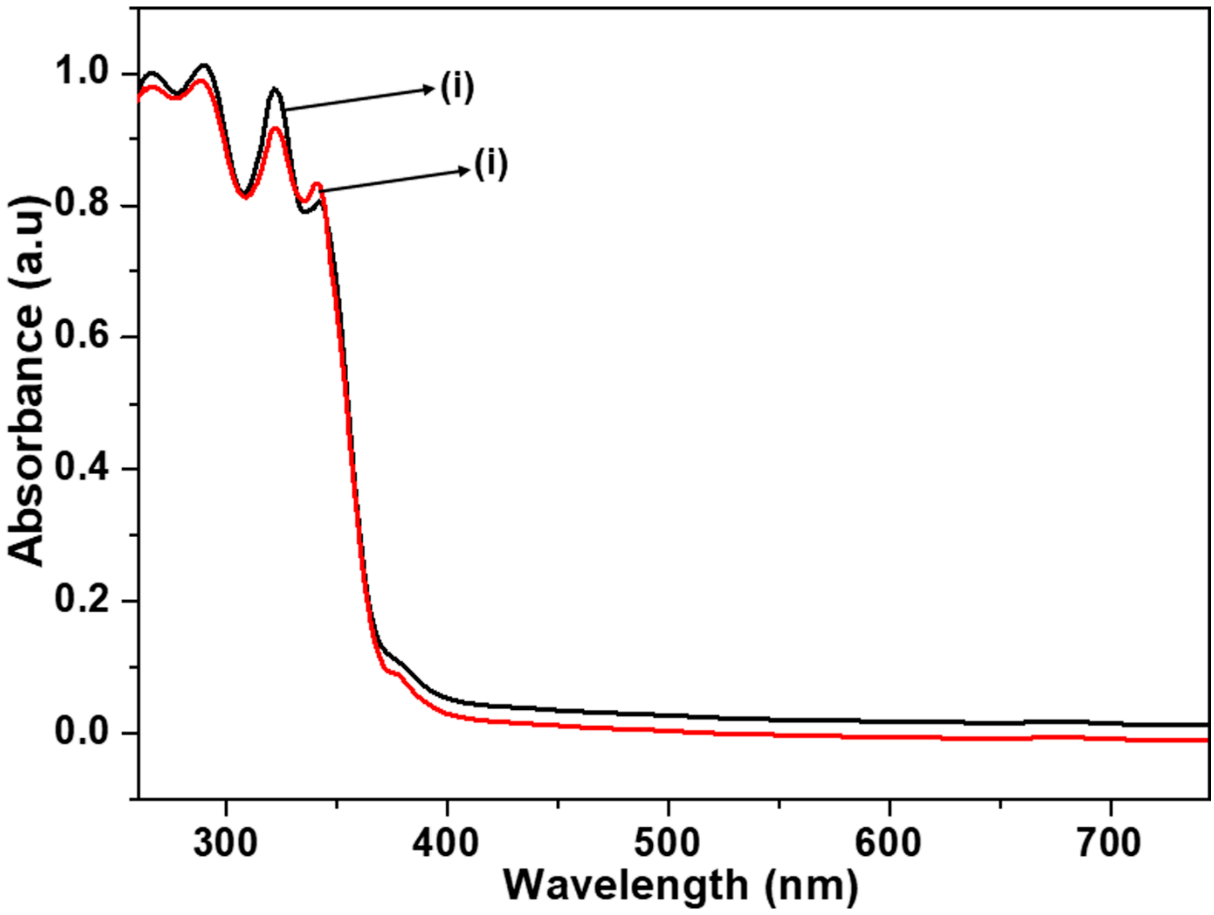


**Figure S2** UV-Vis absorption spectra of red onion leaves extract obtained from (i) Daegu and (ii) Incheon, South Korea.

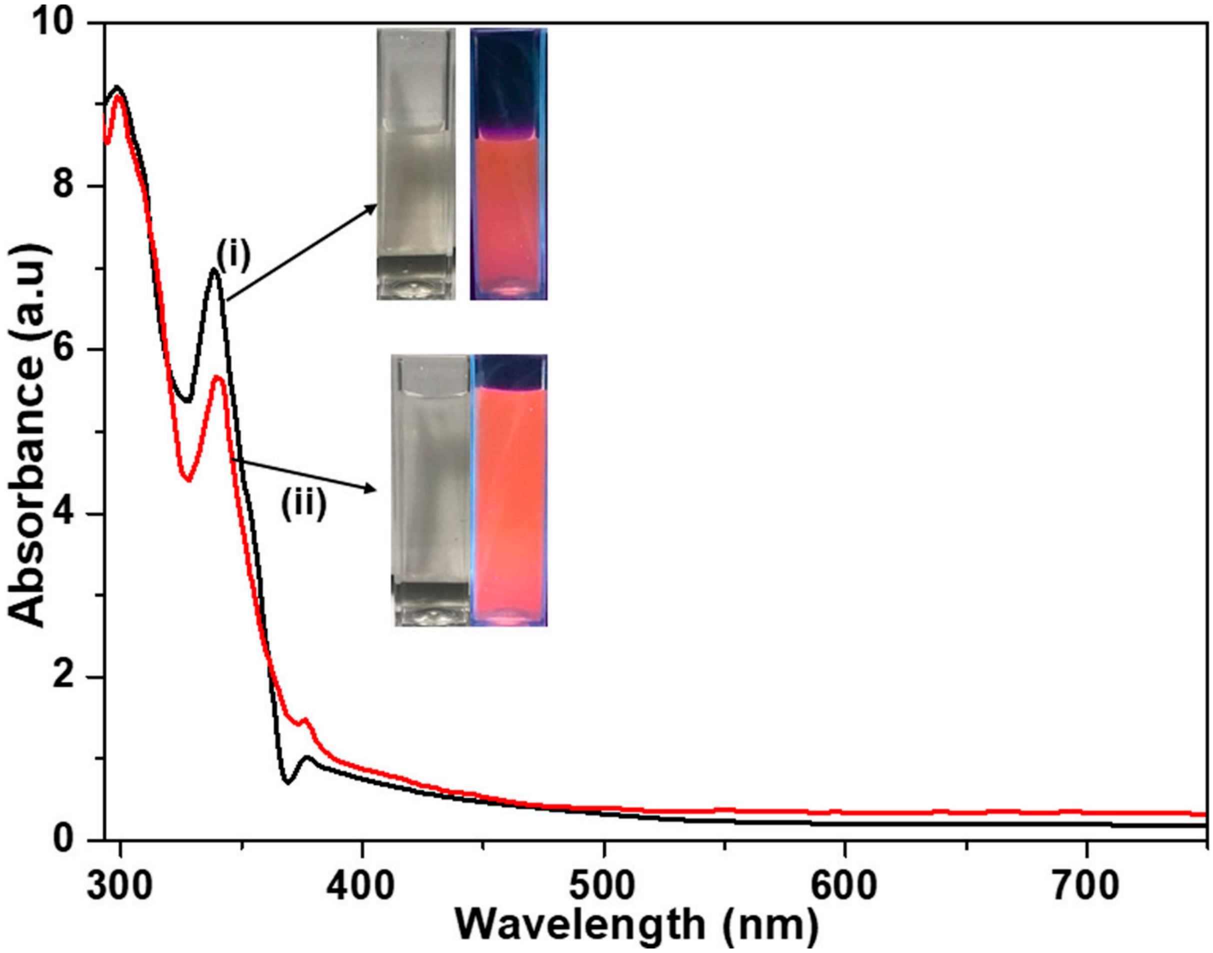


**Figure S3** UV-Vis absorption spectra of GCNDs prepared using by red onion leaves extract obtained from (i) Daegu and (ii) Incheon, South Korea.

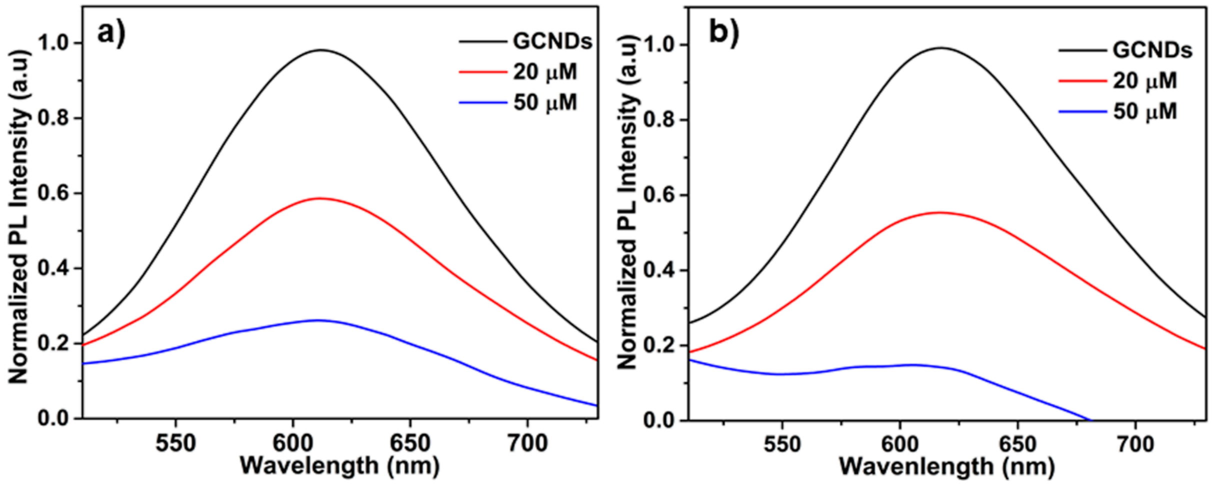


**Figure S4** $Hg^{2+}$ ion sensing by GCNDs prepared using by red onion leaves extract obtained from a) Daegu and b) Incheon, South Korea.
